# Supplementary material for: Genetic analysis of Boletus edulis suggests that intra-specific competition may reduce local genetic diversity as a woodland ages
Source: R Soc Open Sci. 2020 Jul 8;7(7):200419. doi: 10.1098/rsos.200419 (PMC7428248; doi:10.1098/rsos.200419)
Supplement: RMarkdown Code [file rsos200419supp1.pdf]

R-code for 'Mushroom Genotyping Project'

J.I. Hoffman, R. Nagel, V. Litzke, D. A. Wells, W. Amos

- Download packages and libraries
  - Exploring the data
  - Calculating the number of unique genets per population
  - Plotting genet frequency over time
  - Clone Correction
  - Test correlation between genet number and number of sporocarps
  - Plotting spatial autocorrelation obtained using GenAlEx
  - Plotting the relatedness matrix obtained using GenAlEx
  - Calculating sMLH values using `inbreedR`
  - Mean relatedness per individual & sMLH values
  - Mean tree ages

This document provides all the `R code` for the manuscript titled *Genetic analysis of Boletus edulis suggests that intra-specific competition may reduce local genetic diversity as a woodland ages*. Both the R Markdown file and the data can be downloaded from the accompanying via DRYAD (https://datadryad.org/stash/share/t2AOYp64yC-4JOP3cfSZSwRAQRAR49UBCOjZt8wbmH8 (https://datadryad.org/stash/share/t2AOYp64yC-4JOP3cfSZSwRAQRAR49UBCOjZt8wbmH8)). If you have any questions, don't hesitate to contact Joseph Hoffman.

The data originates from samples of the common Steinpilz (*Boletus edulis*) collected from Bielefeld, Germany.

Download packages and libraries

In order to repeat analyses presented in this manuscript a number of packages that extend the functionalities of base `R` are required. These can be installed using the code `install.packages("xxPACKAGENAMExx", dependencies = TRUE)`

```
library(poppr)
library(dplyr)
library(tidyr)
library(ggplot2)
library(ggrepel)
library(viridis)
library(scales)
library(ggpubr)
library(corrplot)
library(inbreedR)
library(readxl)
library(magrittr)
library(grid)
library(gridExtra)
library(AICcmodavg)
library(Matrix)
library(lme4)
library(qvalue)
library(adegenet)
library(reshape2)
library(kableExtra)
library(sjPlot)
library(car)
```

Exploring the data

The datasheet used for the inital set of analyses is a GenAlEx file including individual id, site id, and genotype information at all seven loci.

```
mush <- read.genalex("German cep genotypes_Bielefeld.csv")

# Genotype accumulation curve to determine the minimum number of loci necessary to discrimin
ate between individuals in a population
genotype_curve(mush, sample = 1000, quiet = TRUE)
```

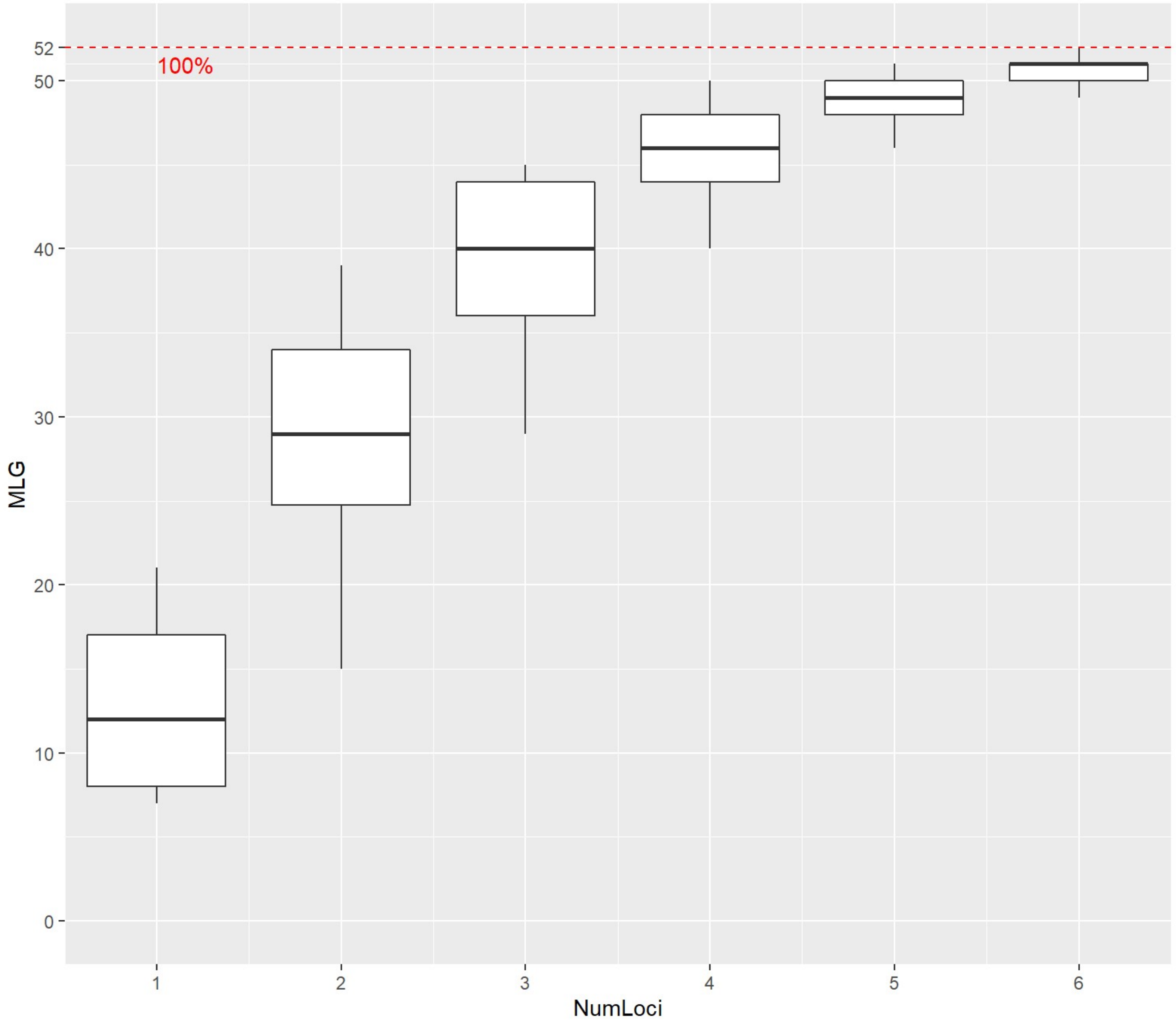


```
# look at missing data & remove individuals missing more than 3 loci
mush %>% missingno("geno", cutoff = 4/nLoc(mush)) %>%  info_table(plot = FALSE)
```

```
##           Locus
## Population  AAC92  AAC71    AC8  AT102  AC111  AC101  ACC81   Mean
##      1          .      .      . 0.0556      .      .      . 0.0079
##      2          .      .      .      .      .      .      .      .
##      3          .      .      .      .      . 0.4000      . 0.0571
##      4     0.0500 0.0500      . 0.1000      .      . 0.2000 0.0571
##      5          .      . 0.2500 0.2500 0.2500      . 0.2500 0.1429
##      6          .      .      .      .      .      .      .      .
##      7          .      .      . 0.3333      .      . 0.3333 0.0952
##      9          . 0.7500      . 0.7500      . 0.2500 1.0000 0.3929
##      10         .      .      .      .      .      .      .      .
##      11         .      .      .      .      .      .      .      .
##      12         . 0.0263 0.0263 0.0263      . 0.0263      . 0.0150
##      13         .      .      .      .      .      .      .      .
##      14         .      .      .      .      .      .      .      .
##      15         .      .      .      .      .      .      .      .
##      Total 0.0075 0.0373 0.0149 0.0672 0.0075 0.0299 0.0746 0.0341
```

Calculating the number of unique genets per population

A genet is a group of genetically identical individuals, whereby an individual refers to the visible fruiting body or sporocarp that develops above ground. Given that fungi can reproduce sexually or clonally, it is important to distinguish between the total number of sporocarps sampled at a given site and the number of unique genets present at that site.

```
# Count number of sporocarps and genets per population
loci <- cbind(data.frame(Pop = mush@strata), data.frame(Genotype = mlg.vector(mush)))
sporocarps <- data.frame(table(loci$Pop))
names(sporocarps) <- c("OriginalPopNr", "sporocarps")
genets <- data.frame(loci %>% group_by(Pop) %>% summarise(count = n_distinct(Genotype)))
names(genets) <- c("OriginalPopNr", "genets")
SporGen <- left_join(sporocarps, genets, by="OriginalPopNr")

SporGen
```

```
##    OriginalPopNr sporocarps genets
## 1              1         18      3
## 2              2          8      1
## 3              3          5      5
## 4              4         20     14
## 5              5          4      4
## 6              6         14      4
## 7              7          3      3
## 8              9          4      4
## 9             10          4      3
## 10            11          6      4
## 11            12         38      6
## 12            13          2      1
## 13            14          7      1
## 14            15          1      1
```

```
# Count frequency distribution of genets per population
genets.count <- data.frame(loci %>% group_by(Pop) %>% count(Genotype))
names(genets.count) <- c("OriginalPopNr", "Genotype", "Freq")
```

```
# reorder populations by genet frequency
loci <- inner_join(genets.count, SporGen, by="OriginalPopNr")
loci <- loci[with(loci, order(-sporocarps, -Freq)),]
loci$OriginalPopNr <- factor(loci$OriginalPopNr, levels=c("12","4","1","6","2","14","11","
3","5","9","7","10","13","15"))
loci$GenotypeNrNew <- 1:54
```

```
# plot
ggplot(aes(x = OriginalPopNr,
           y = Freq,
           fill = GenotypeNrNew),
       data = loci) +
  geom_bar(stat = "identity", color = "black") +
  labs(x = "sample site", y = "no. sporocarps") +
  scale_y_continuous(expand = c(0, 0), limits = c(0,40)) +
  scale_x_discrete(labels = c(1:15)) +
  scale_fill_viridis(name = "Genotype", option="viridis", direction = -1, begin = 0.13) +
  theme(panel.grid.major = element_blank(), panel.grid.minor = element_blank(),
        panel.background = element_blank(), axis.line = element_line(colour = "black"),
        text = element_text(size = 15),
        axis.text.x = element_text(vjust=1))
```

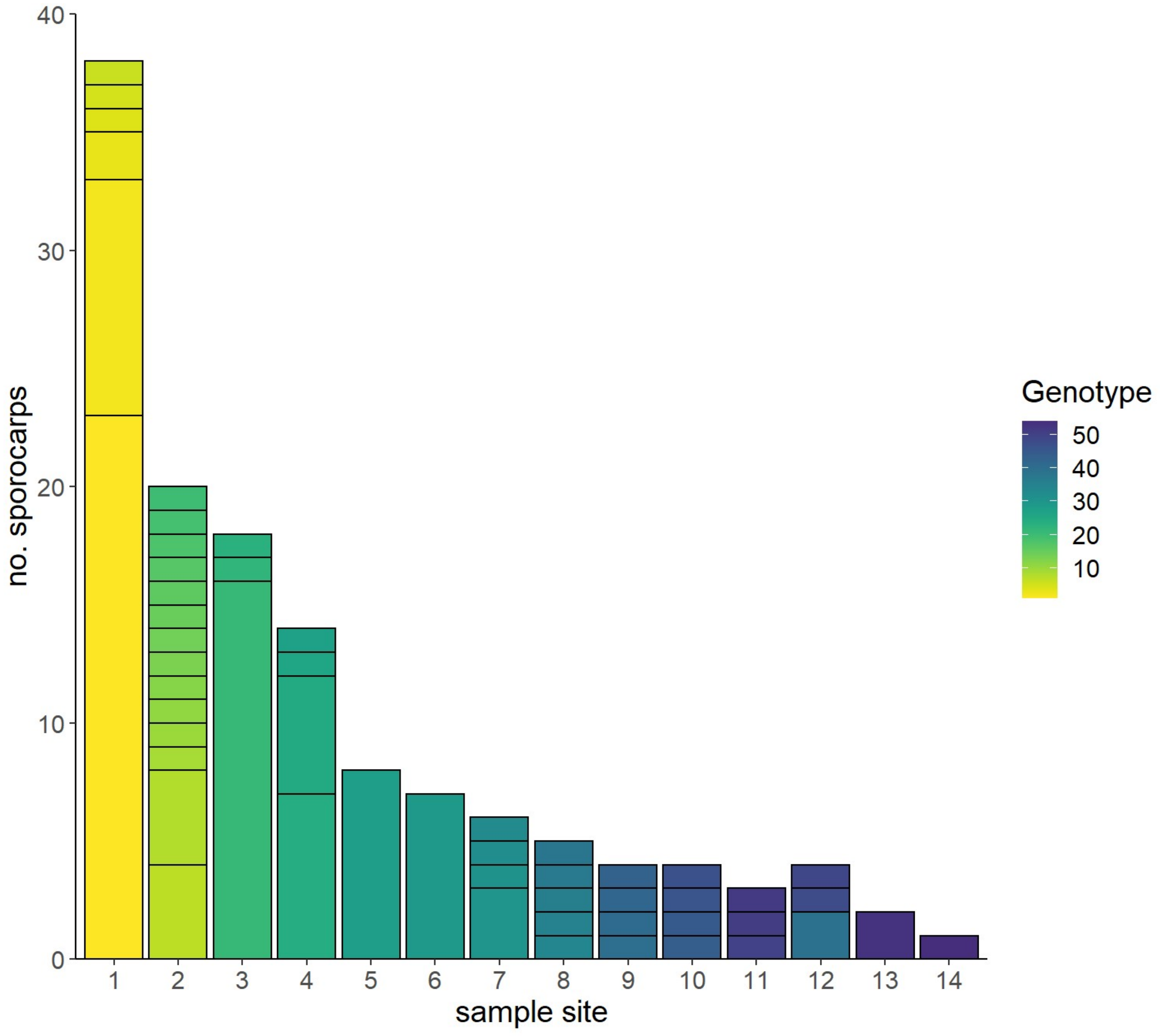

Plotting genet frequency over time

Sites at which sporocarps were collected at multiple time points are shown here. This gives an indication of how genet frequency varies per site over time.

```
added <- read.table("German cep genotypes 28_08_19_MoreInfo.csv", header=TRUE, sep=",")
added$DateSampled <- as.Date(added$DateSampled, "%d-%m-%y")
head(added)
```

```
##   Indiv Pop_old Pop_new Pop_name DateSampled Genotype Genotype1 Frequency
## 1  Bi70      12       1 hallowed  2015-09-20        8         1        10
## 2  Bi71      12       1 hallowed  2015-09-20        8         1        10
## 3  Bi72      12       1 hallowed  2015-09-20        8         1        10
## 4  Bi73      12       1 hallowed  2015-09-20        8         1        10
## 5  Bi74      12       1 hallowed  2015-09-20        8         1        10
## 6  Bi75      12       1 hallowed  2015-09-20        8         1        10
##   AAC92 X AAC71 X.1 AC8 X.2 AT102 X.3 AC111 X.4 AC101 X.5 ACC81 X.6
## 1     3 3     3   3   2   3     4   4    24  24    10  11     8  12
## 2     3 3     3   3   2   3     4   4    24  24    10  11     8  12
## 3     3 3     3   3   2   3     4   4    24  24    10  11     8  12
## 4     3 3     3   3   2   3     4   4    24  24    10  11     8  12
## 5     3 3     3   3   2   3     4   4    24  24    10  11     8  12
## 6     3 3     3   3   2   3     4   4    24  24    10  11     8  12
```

```
added.1 <- unique(data.frame(added$Pop_new,added$Genotype1, added$Frequency,
                             added$DateSampled))
added.1 <- subset(added.1, added.Pop_new %in% c("1", "3", "4", "5", "7", "12"))

added.1$order = factor(added.1$added.Pop, levels=c("1", "3", "4", "5", "7", "12"))

labels <- c("1" = "Site 1", "3" = "Site 3", "4" = "Site 4", "5" = "Site 5",
            "7" = "Site 7", "12" = "Site 12")
integer_breaks <- function(n = 5, ...) {
  breaker <- pretty_breaks(n, ...)
  function(x) {
    breaks <- breaker(x)
    breaks[breaks == floor(breaks)]
  }
}
```

```
ggplot(aes(x = added.DateSampled, y = added.Frequency, fill = added.Genotype1),
       data = added.1) +
  geom_bar(stat = "identity", color = "black", width = 1) +
  labs(x = "Date sampled", y = "Genotype frequency") +
  scale_x_date(breaks = pretty_breaks(15)) +
  scale_fill_viridis(name = "Genotype", option="viridis", direction = -1, begin = 0.13) +
  theme_classic() +
  theme(panel.grid.major = element_blank(),
        panel.grid.minor = element_blank(),
        panel.background = element_blank(),
        axis.line.x = element_blank(),
        text = element_text(size = 15),
        strip.text.y = element_blank(),
        panel.spacing.y = unit(8, "mm"),
        axis.text.x=element_text(angle = 45, hjust = 1)) +
  geom_hline(yintercept=0) +
  scale_y_continuous(expand = c(0, 0), breaks = integer_breaks()) +
  facet_grid(order ~ ., labeller=labeller(order = labels), scale = "free_y")
```

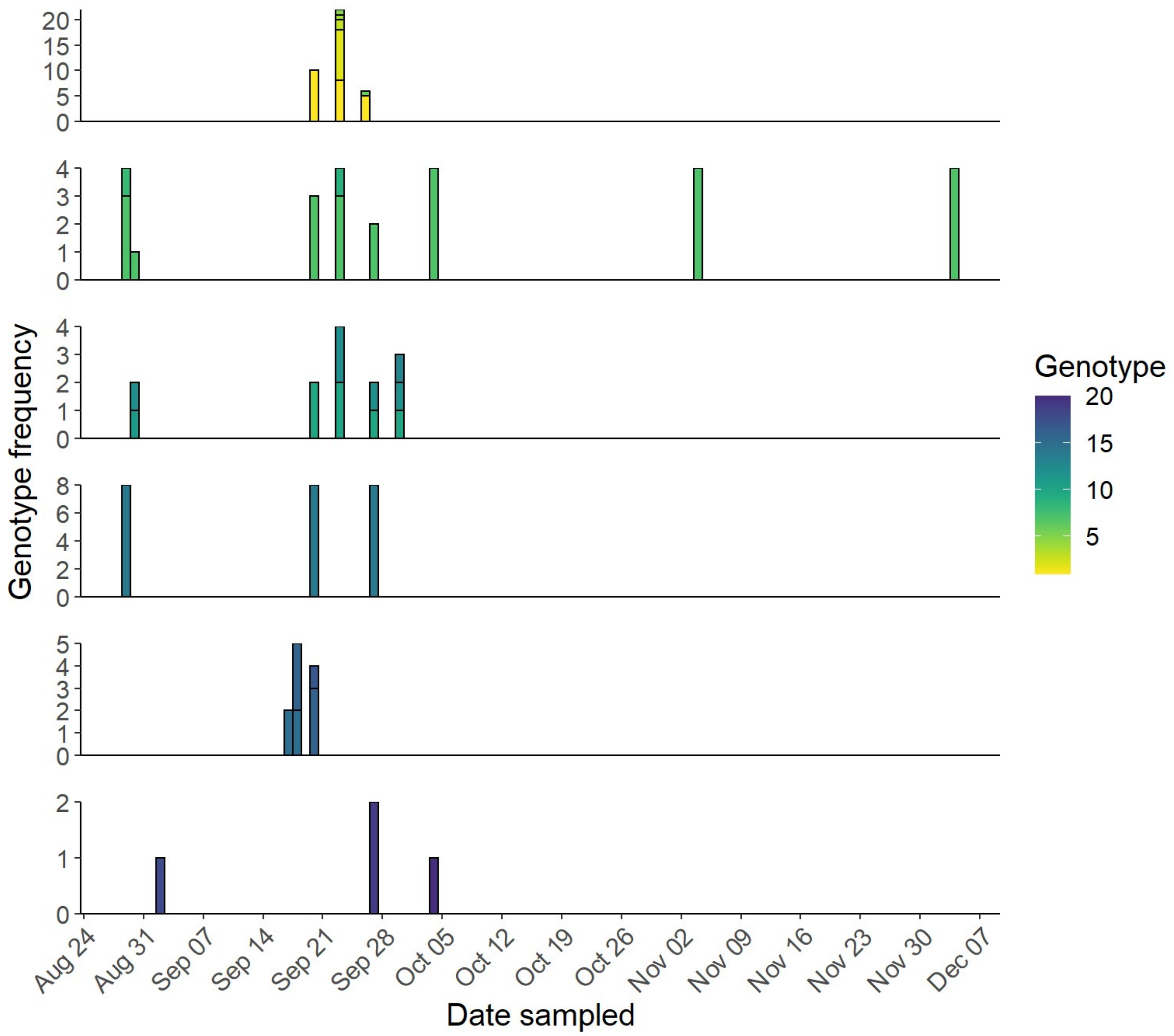

Clone Correction

The `poppr` package is useful for the analysis of genetic data originating from systems with mixed modes of reproduction (i.e. sexual and clonal reproduction) [1], [2]. We strongly recommend the poppr primer (http://grunwaldlab.github.io/Population_Genetics_in_R/Data_Preparation.html) for an introduction to the package.

```
# index of associaiton = clonal populations are identified by an IA value that differs
# significantly from zero.
ia(mush, sample = 999)
```

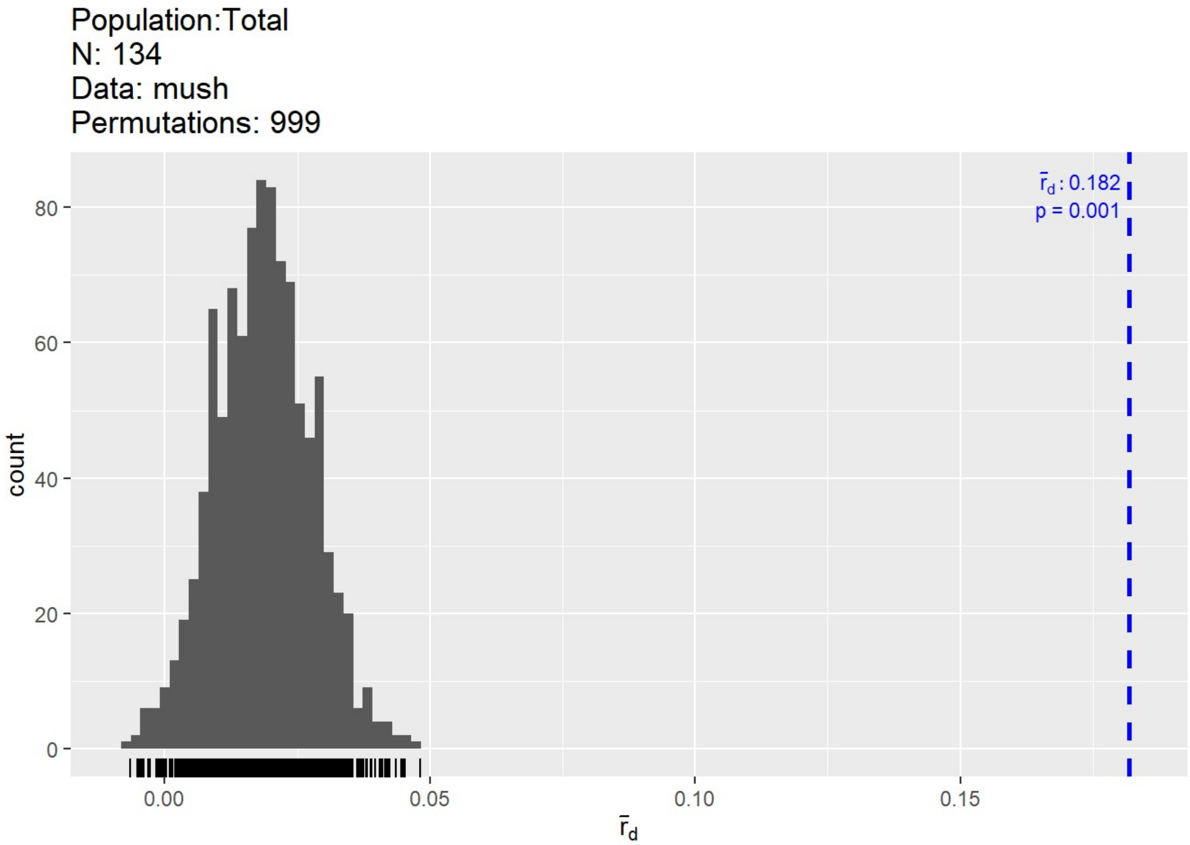


```
##        Ia      p.Ia     rbarD      p.rD
## 1.0621056 0.0010000 0.1818518 0.0010000
```

```
# clone correction = remove potential bias caused by cloned genotypes
mcc <- clonecorrect(mush)
ia(mcc, sample = 999)
```

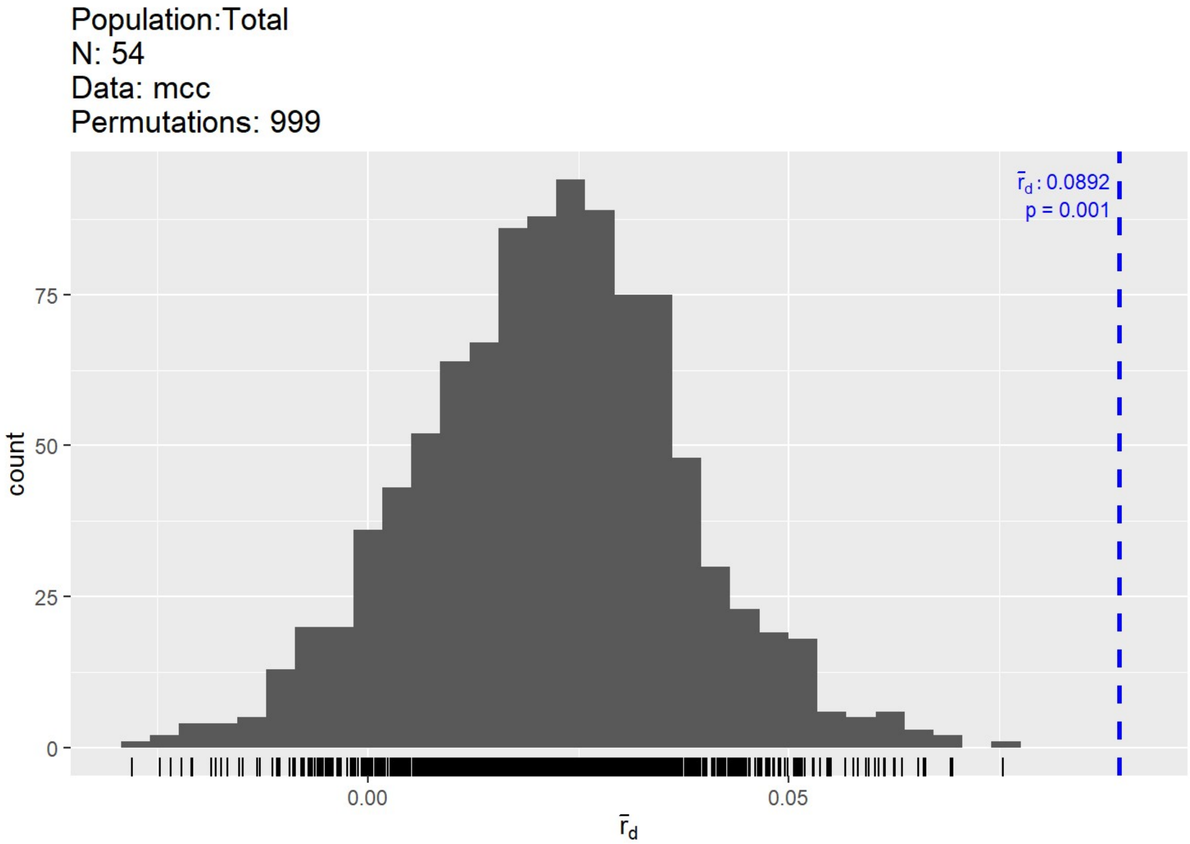

```
##          Ia       p.Ia      rbarD       p.rD
## 0.51683488 0.00100000 0.08922348 0.00100000
```

```
# locus table
locus_table(mcc, info = FALSE)
```

```
##        summary
## locus   allele   1-D  Hexp Evenness
##   AAC92   6.00  0.75  0.76     0.92
##   AAC71   7.00  0.69  0.70     0.70
##   AC8     6.00  0.52  0.52     0.63
##   AT102   5.00  0.61  0.61     0.67
##   AC111  12.00  0.75  0.76     0.56
##   AC101  11.00  0.77  0.78     0.63
##   ACC81   5.00  0.19  0.19     0.40
##   mean    7.43  0.61  0.62     0.64
```

```
# Calculating genotypic diversity
poppr(mcc)
```

```
##      Pop  N MLG  eMLG    SE    H    G lambda  E.5  Hexp    Ia  rbarD File
## 1      1  3   3  3.00 0.000 1.10  3.0  0.667 1.00 0.467 3.571 1.0000  mcc
## 2      2  1   1  1.00 0.000 0.00  1.0  0.000  NaN 0.571    NA     NA  mcc
## 3      3  5   5  5.00 0.000 1.61  5.0  0.800 1.00 0.425 0.672 0.1703  mcc
## 4      4 14  14 10.00 0.000 2.64 14.0  0.929 1.00 0.423 0.278 0.0594  mcc
## 5      5  4   4  4.00 0.000 1.39  4.0  0.750 1.00 0.427 0.333 0.1667  mcc
## 6      6  4   4  4.00 0.000 1.39  4.0  0.750 1.00 0.520 2.358 0.5051  mcc
## 7      7  3   3  3.00 0.000 1.10  3.0  0.667 1.00 0.410 1.167 0.7000  mcc
## 8      9  4   4  4.00 0.000 1.39  4.0  0.750 1.00 0.310 0.163 0.0965  mcc
## 9     10  3   3  3.00 0.000 1.10  3.0  0.667 1.00 0.657 0.400 0.1000  mcc
## 10    11  4   4  4.00 0.000 1.39  4.0  0.750 1.00 0.418 0.545 0.1014  mcc
## 11    12  6   6  6.00 0.000 1.79  6.0  0.833 1.00 0.722 1.651 0.2869  mcc
## 12    13  1   1  1.00 0.000 0.00  1.0  0.000  NaN 0.857    NA     NA  mcc
## 13    14  1   1  1.00 0.000 0.00  1.0  0.000  NaN 0.429    NA     NA  mcc
## 14    15  1   1  1.00 0.000 0.00  1.0  0.000  NaN 0.286    NA     NA  mcc
## 15 Total 54  52  9.94 0.245 3.94 50.3  0.980 0.98 0.617 0.517 0.0892  mcc
```

Test correlation between genet number and number of sporocarps

```
genets <- data.frame(Pop = c(1:14),
                     NrSporocarps = c(38,20,18,14,8,7,6,5,4,4,3,4,2,1),
                     NrGenets = c(6,14,3,4, 1,1,4,5,4,4,3,3,1,1))

cor.test(genets$NrGenets, genets$NrSporocarps)
```

```
##
##  Pearson's product-moment correlation
##
## data:  genets$NrGenets and genets$NrSporocarps
## t = 2.0443, df = 12, p-value = 0.06351
## alternative hypothesis: true correlation is not equal to 0
## 95 percent confidence interval:
##  -0.03060276  0.81818112
## sample estimates:
##       cor
## 0.508229
```

```
ggscatter(genets, x = "NrGenets", y = "NrSporocarps",
          add = "reg.line", conf.int = TRUE,
          cor.coef = TRUE, cor.method = "pearson",
          xlab = "Number of Genets", ylab = "Number of Sporocarps")
```

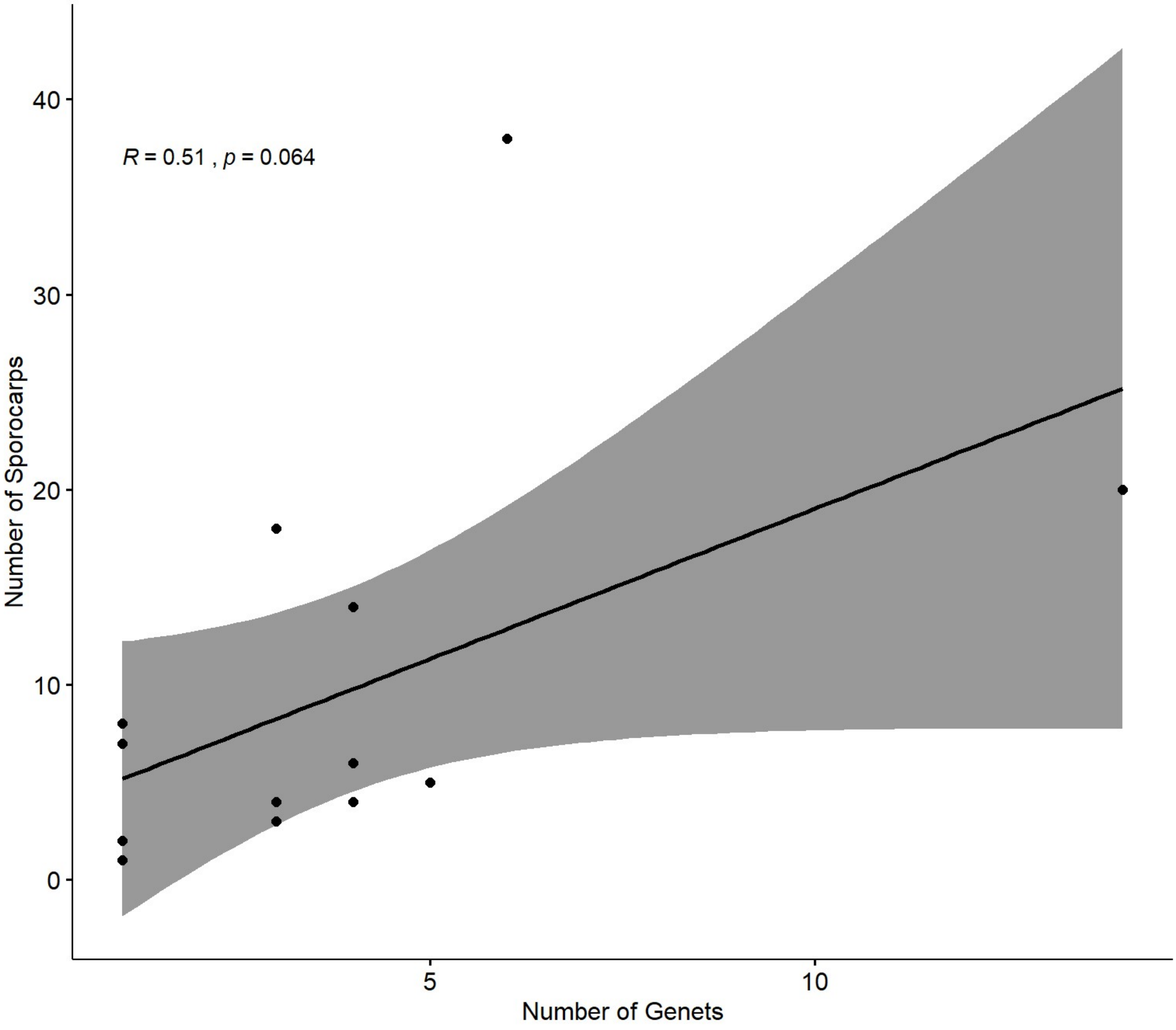

Plotting spatial autocorrelation obtained using GenAlEx

Spatial autocorrelation anaylsis was done at a distance class of 250m with the number of permutations and bootstraps set to 1000 using GenAlEx version 6.5 [3]. The software is freely available (http://bioinformatics.oxfordjournals.org/content/28/19/2537) and helpful tutorials can be found online (http://biology-assets.anu.edu.au/GenAlEx/Tutorials.html).

```
twofifty <- read.table("SpatialAutocorrelation_250m.csv",
                       header = TRUE, sep = ",")

ggplot(data = twofifty[1:27,],
       aes(x=DistanceClass, y=r, ymin=Ur, ymax=Lr)) +
  geom_pointrange(color="black", fill="black", shape=19, size=0.5) +
  geom_line(aes(y=U), color = "#55C667FF", linetype="dashed", size = 1) +
  geom_line(aes(y=L), color = "#55C667FF", linetype="dashed", size = 1) +
  geom_line(aes(y=0)) +
  scale_x_continuous(breaks = pretty(twofifty$DistanceClass, n = 25)) +
  labs(x = "\nDistance (km)",
       y = expression(paste("Genetic correlation (", italic("r"),")"))) +
  theme_classic() +
  theme(text = element_text(size = 15),
        axis.text.x=element_text(vjust=0.2))
```

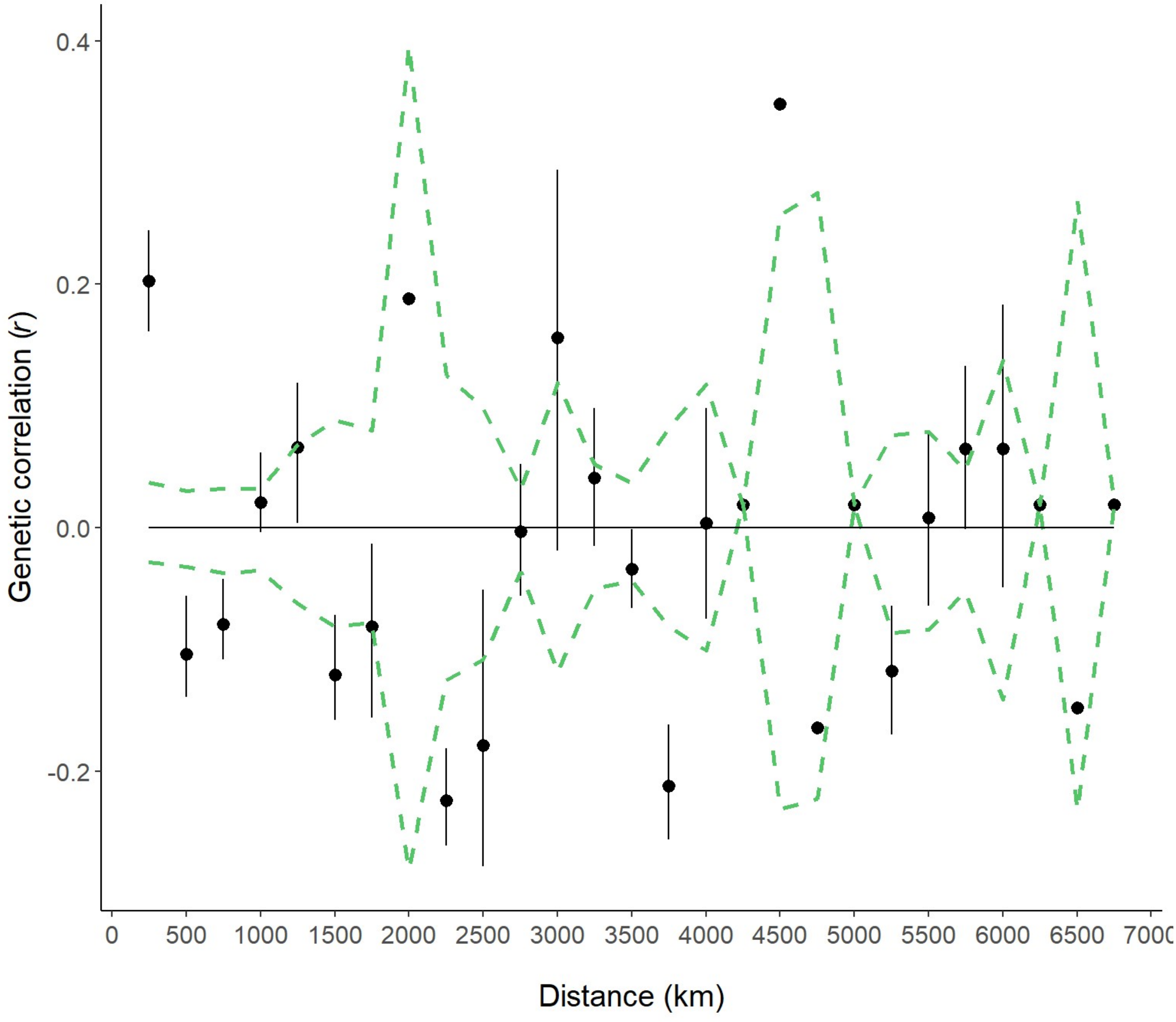

Plotting the relatedness matrix obtained using GenAlEx

Pairwise relatedness was estimated according to Lynch & Ritland (1999)[4] using GenAlEx version 6.5 (please see above). Results are visuallized here using `corrplot` [5].

```
# individual pairwise
relate <- read.table("Lynch_Ritland_1999_Related_Poprenamed_Bielefeld1-54.csv",
                     header=TRUE, row.names = 1, sep=",", check.names=FALSE)

corrplot(as.matrix(relate), type="upper", method="color", is.corr = FALSE, tl.pos = "td",
         tl.col="black", col = viridis(100, direction = -1, begin = 0.13))
```

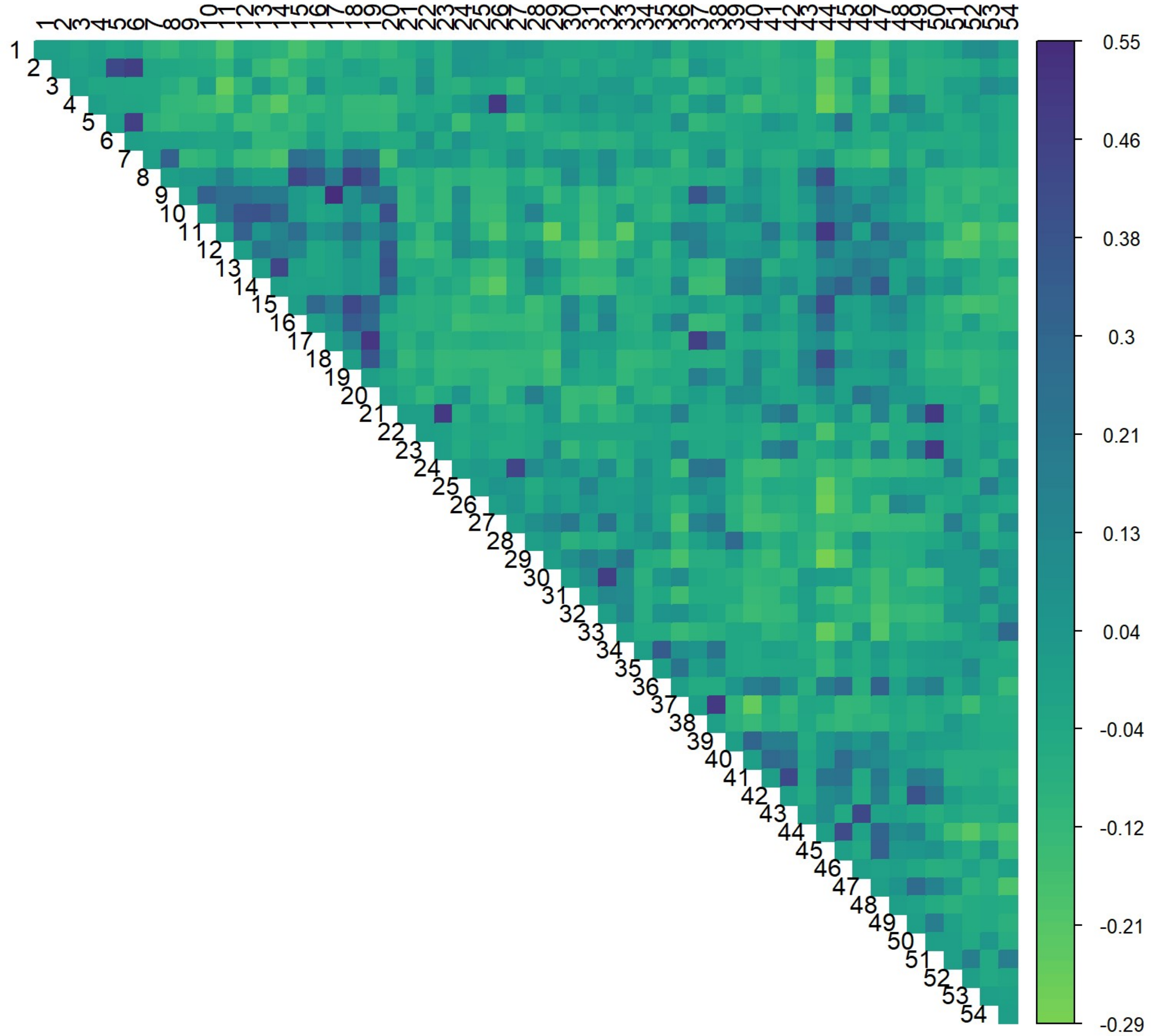


```
# population means
relate.pop <- read.table("Lynch_Ritland_1999_Related_Poprenamed_PopMean_Bielefeld.csv",
                         header=TRUE, sep=",")

relate.pop$Pop <- factor(relate.pop$Pop, levels = relate.pop$Pop)

ggplot(data = relate.pop,
       aes(x=as.factor(Pop), y=Mean, ymin=Uv, ymax=Lv)) +
  geom_pointrange(color="black", fill="white", shape=22) +
  labs(x = "Population", y = "Mean Relatedness",
       title = "Within Population Pairwise Values",
       subtitle = "Lynch & Ritland (1999)") +
  theme_classic() +
  theme(axis.text.x = element_text(angle = 45, hjust = 1),
        text = element_text(size = 10))
```

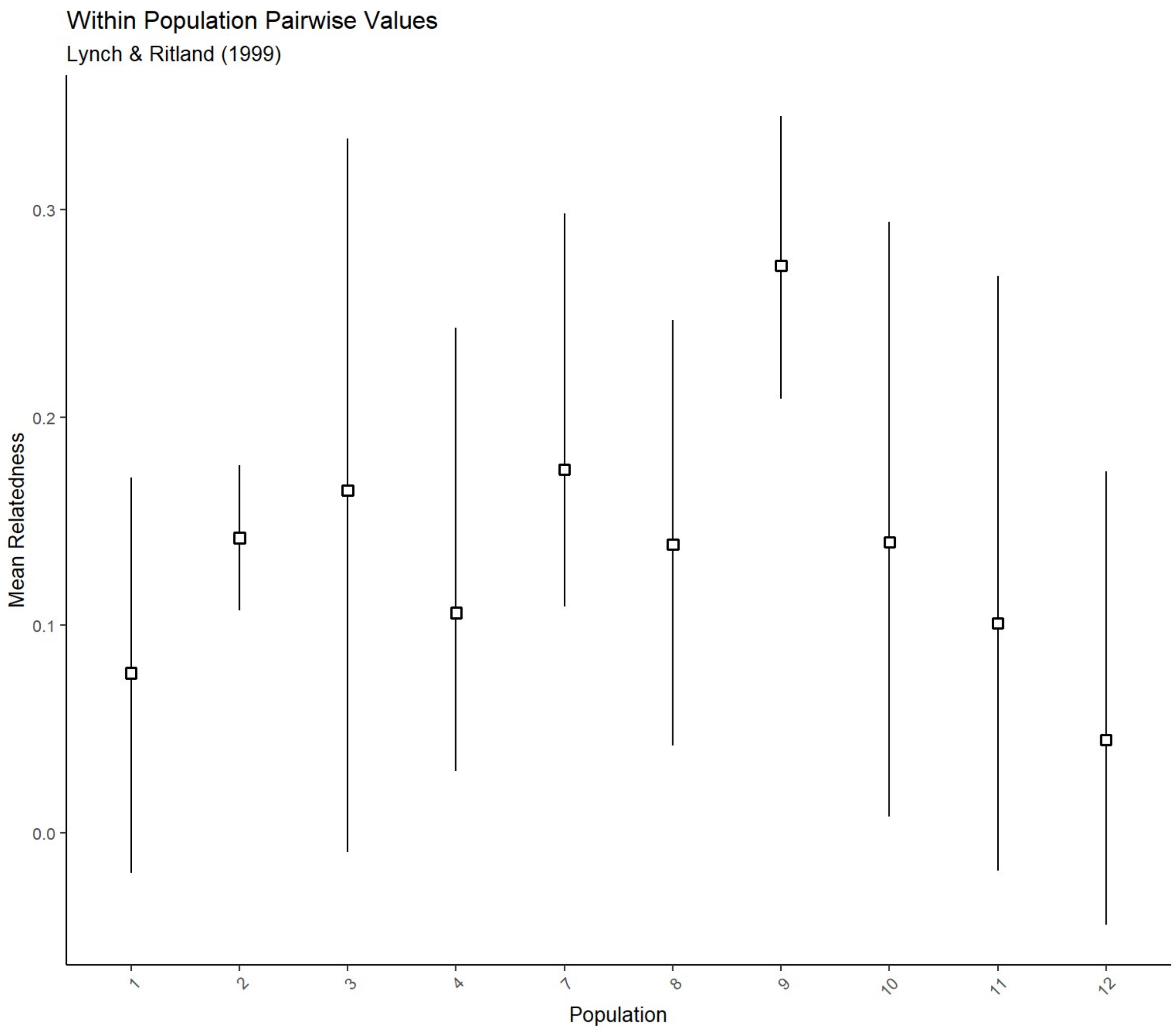

Calculating sMLH values using `inbreedR`

In order to use `inbreedR` [6], the working format is typically an *individual x loci* matrix, where rows represent individuals and every two columns represent a single locus. If an individual is heterozygous at a given locus, it is coded as 1, whereas a homozygote is coded as 0, and missing data are coded as NA.

```
# read data
mushroom <- readxl::read_xlsx("data.xlsx")[1:55, ]
# express alleles as numerals
mushroom[3:ncol(mushroom)] <- lapply(mushroom[3:ncol(mushroom)], as.numeric)
head(mushroom)
```

```
## # A tibble: 6 x 16
##   ID    Population AAC92.a AAC92.b AAC71.a AAC71.b AC8.a AC8.b AT102.a
##   <chr>      <dbl>   <dbl>   <dbl>   <dbl>   <dbl> <dbl> <dbl>   <dbl>
## 1 Bi70           1       3       3       3       3     2     3       4
## 2 Bi85           1       3       3       3       6     2     4       4
## 3 Bi86           1       5       5       3       6     2     2       4
## 4 Bi93           1       3       3       7       3     2    12       3
## 5 Bi97           1       2       2       0       0     0     0       0
## 6 Bi113          1       2       3       3       6     2     4       4
## # ... with 7 more variables: AT102.b <dbl>, AC111.a <dbl>, AC111.b <dbl>,
## #   AC101.a <dbl>, AC101.b <dbl>, ACC81.a <dbl>, ACC81.b <dbl>
```

Since demographic data is present in the beginning of our data frame, we will start our new genotype file from the 3rd column onwards. The function `convert_raw` converts a common format for genetic markers (two columns per locus) into the `inbreedR` working format. Afterwards, `check_data` allows us to test whether the genotype data frame has the correct format for subsequent analyses that use `inbreedR` functions.

```
mushroom_geno <- convert_raw(mushroom[3:ncol(mushroom)])
invisible(lapply(mushroom_geno, table, useNA = "always"))
check_data(mushroom_geno, num_ind = 55, num_loci = 7)
```

```
# Create Dataframe & Estimate heterozygosity

# estimate heterozygosity
het <- sMLH(mushroom_geno)

# change dataframe
mushdf <- data.frame(ID = mushroom[[1]], Population = mushroom[[2]])
#mushdf_reshaped <- reshape2::melt(mushdf)
```

Mean relatedness per individual & sMLH values

```
mlh <- read.table("sMLH_r_230919.csv", header = TRUE, sep = ",")
mlh$Population <- as.factor(mlh$Population)
head(mlh)
```

```
##      ID Population       het      mean.r
## 1  Bi70          1 0.9700599 -0.04732684
## 2  Bi85          1 1.9401198 -0.03014350
## 3  Bi86          1 1.2934132 -0.04564737
## 4  Bi93          1 1.2934132 -0.04317690
## 5  Bi97          1 0.9700599 -0.02722012
## 6 Bi113          1 1.9401198 -0.02000333
```

```
#significant differences in relatedness values among the study sites?
shapiro.test(mlh$mean.r)
```

```
##
##  Shapiro-Wilk normality test
##
## data:  mlh$mean.r
## W = 0.93566, p-value = 0.006171
```

```r
#data is not normally distributed -> Kruskal-Wallis Test
kruskal.test(mean.r ~ Population, data = mlh)
```

```
##
##  Kruskal-Wallis rank sum test
##
## data:  mean.r by Population
## Kruskal-Wallis chi-squared = 41.352, df = 13, p-value = 8.354e-05
```

```r
#significant differences in individual sMLH among the study sites?
shapiro.test(mlh$het)
```

```
##
##  Shapiro-Wilk normality test
##
## data:  mlh$het
## W = 0.9341, p-value = 0.005329
```

```r
#data is not normally distributed -> Kruskal-Wallis Test
kruskal.test(het ~ Population, data = mlh)
```

```
##
##  Kruskal-Wallis rank sum test
##
## data:  het by Population
## Kruskal-Wallis chi-squared = 23.024, df = 13, p-value = 0.04139
```

```r
#r and sMLH correlated?
cor.test(mlh$het, mlh$mean.r)
```

```
##
##  Pearson's product-moment correlation
##
## data:  mlh$het and mlh$mean.r
## t = -2.5274, df = 52, p-value = 0.01457
## alternative hypothesis: true correlation is not equal to 0
## 95 percent confidence interval:
##  -0.54982112 -0.06911481
## sample estimates:
##        cor
## -0.3307544
```

```r
ggscatter(mlh, x = "mean.r", y = "het",
          add = "reg.line", conf.int = TRUE,
          cor.coef = TRUE, cor.method = "pearson",
          xlab = expression(italic("r")), ylab = "sMLH")
```

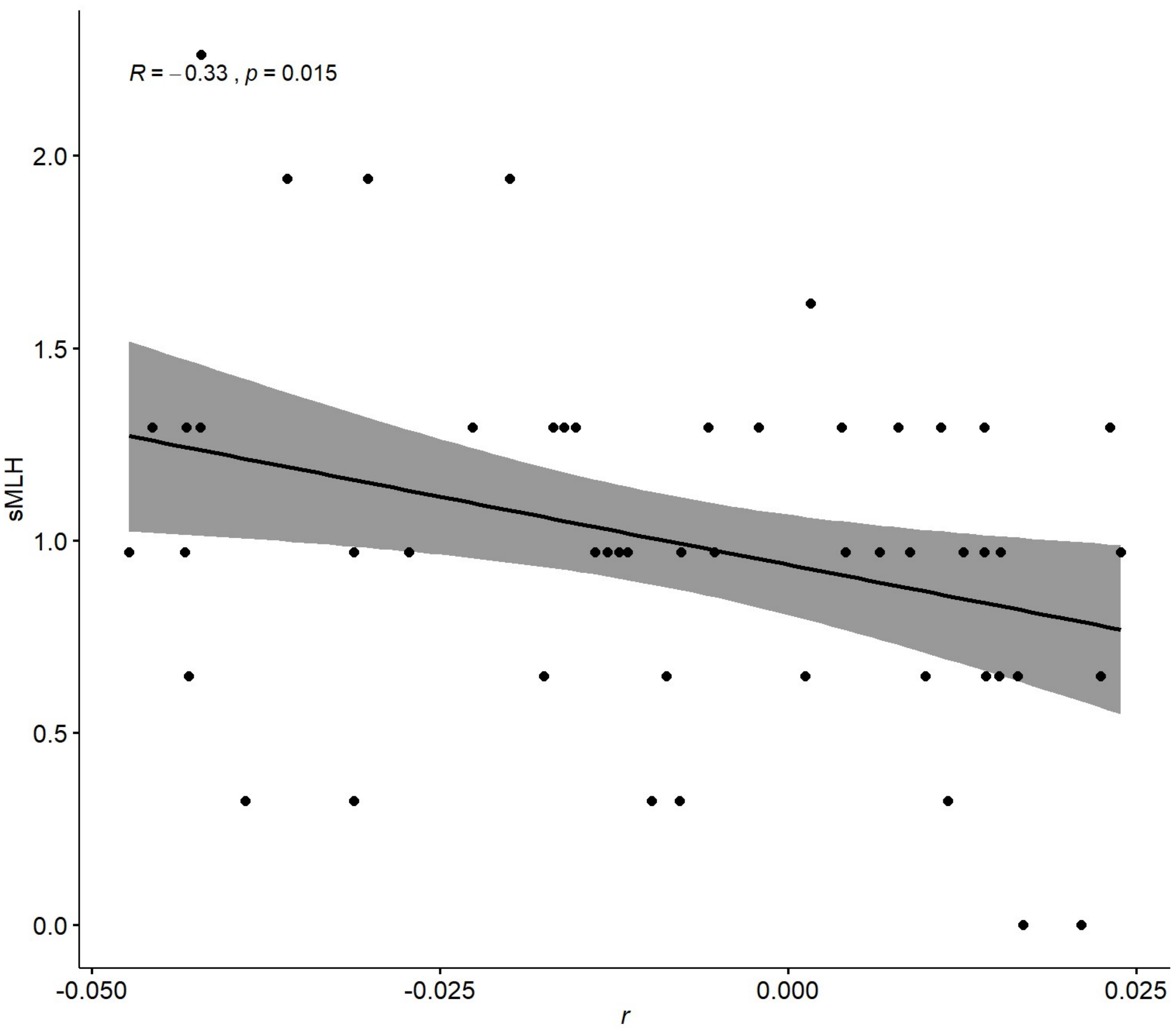

Mean tree ages

The diameter of all trees within a given site area was used to assess the age of the trees. The average age of all trees found at a given site is then used a proxy for site/ woodland age.

```
tmeans <- read.table("TreeDiameters_MeanPerSite.csv", header = TRUE, sep = ",")
head(tmeans)
```

```
##     MeanAge GenetsNr SporocarpsNr Site Shannon      sMLH     r   He
## 1  76.07143        6           38    1    1.79 1.4011976 0.077 0.72
## 2  23.33333       14           20    2    2.64 0.8314799 0.142 0.42
## 3  50.04545        3           18    3    1.10 0.9700599 0.165 0.47
## 4  72.62500        4           14    4    1.39 0.9700599 0.106 0.52
## 5  70.60000        1            8    5    0.00 1.2934132 0.000 0.57
## 6 189.14286        1            7    6    0.00 0.9700599 0.000 0.43
##   NrTrees Simpson MainSpecies
## 1      42    0.83       Beech
## 2     108    0.93       Beech
## 3      22    0.67       Beech
## 4      24    0.75       Beech
## 5      15    0.00       Beech
## 6       7    0.00         Oak
```

```r
# build model for relationship between age of sites and weighted number of genets

hist(cbind(tmeans$GenetsNr, tmeans$SporocarpsNr-tmeans$GenetsNr),
     main = "", xlab = "Number of genets weighted by number of sporocarps")
```

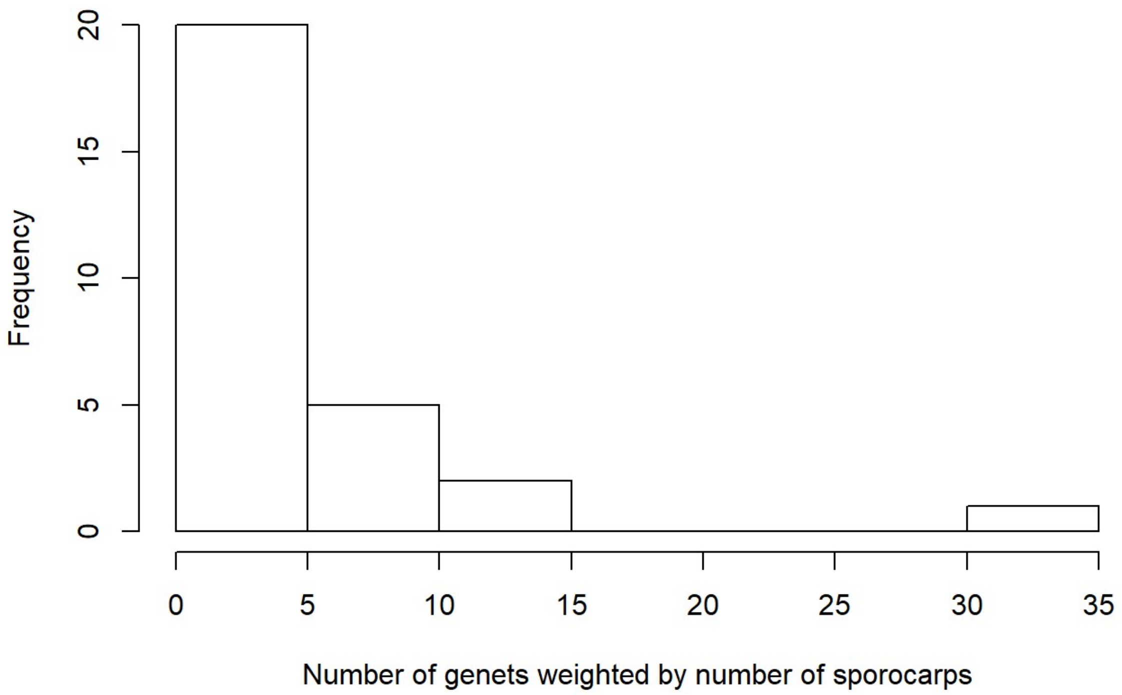


```r
m1 <- glm(formula = GenetsNr/SporocarpsNr ~ MeanAge, family = poisson(),
          data = tmeans, weights = SporocarpsNr)
summary(m1)
```

```
##
## Call:
## glm(formula = GenetsNr/SporocarpsNr ~ MeanAge, family = poisson(),
##     data = tmeans, weights = SporocarpsNr)
##
## Deviance Residuals:
##     Min       1Q   Median       3Q      Max
## -2.0856  -0.1703   0.7267   1.2364   1.7705
##
## Coefficients:
##              Estimate Std. Error z value Pr(>|z|)
## (Intercept) -0.062510   0.322836  -0.194  0.84647
## MeanAge     -0.014495   0.005548  -2.612  0.00899 **
## ---
## Signif. codes:  0 '***' 0.001 '**' 0.01 '*' 0.05 '.' 0.1 ' ' 1
##
## (Dispersion parameter for poisson family taken to be 1)
##
##     Null deviance: 30.840  on 13  degrees of freedom
## Residual deviance: 22.033  on 12  degrees of freedom
## AIC: Inf
##
## Number of Fisher Scoring iterations: 5
```

```
ggplot(data = tmeans, aes(x=MeanAge, y=GenetsNr/SporocarpsNr, weight = SporocarpsNr)) +
  geom_point(aes(color= factor(Site), size= SporocarpsNr)) +
  scale_size_continuous(range = c(2, 8)) +
  geom_text_repel(aes(label=factor(Site)), segment.color = NA, box.padding = 0.01) +
  scale_color_viridis(discrete=TRUE, direction = -1, begin = 0.13) +
  theme_classic() +
  theme(text = element_text(size = 15),
        legend.position = "none") +
  labs(y = "Genets / sporocarps", x = "Mean age (years)") +
  geom_smooth(method = "glm", se = TRUE,
              method.args = list(family = "poisson"), color = "black")
```

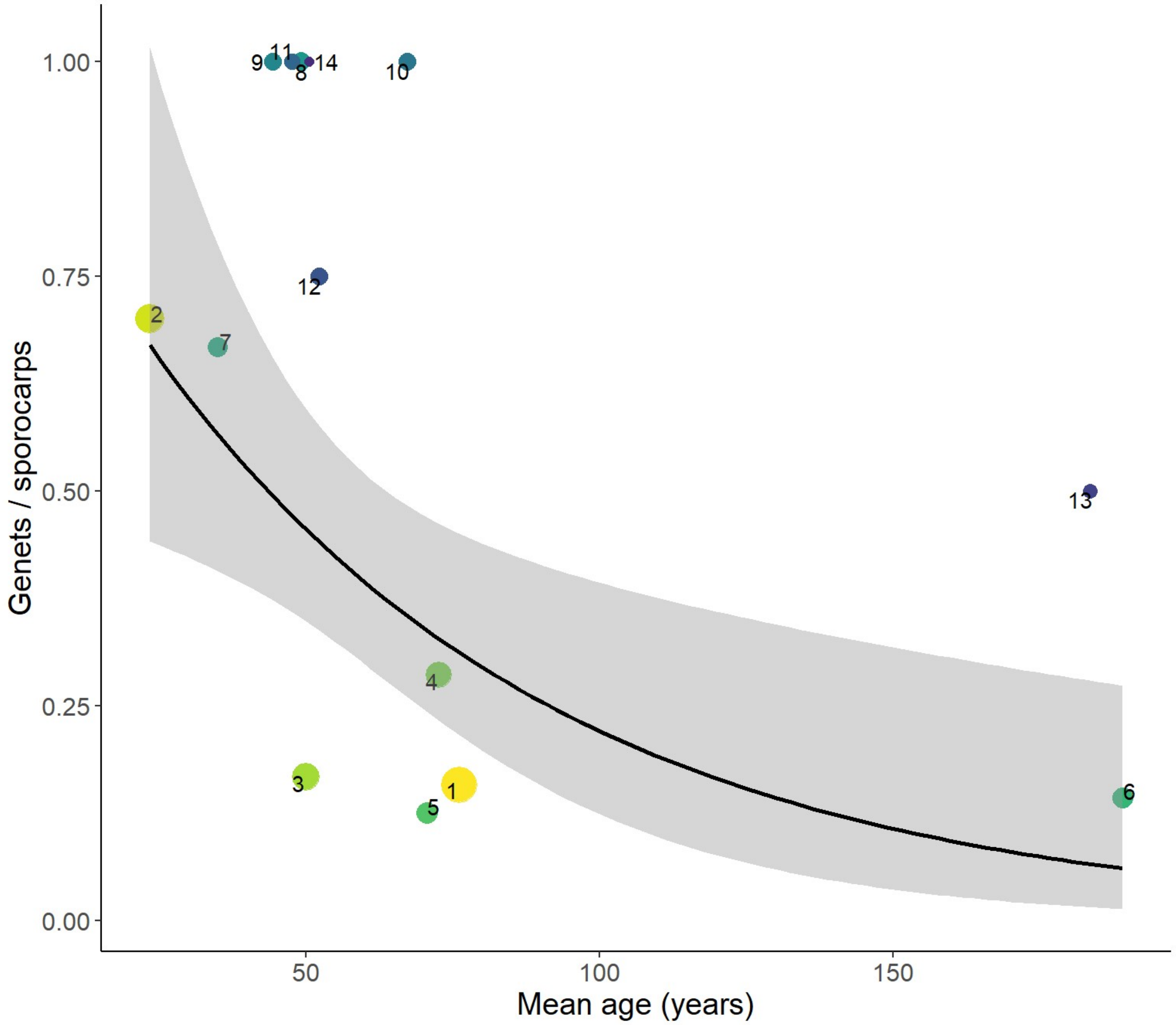


```
# build model for relationship between age of sites and weighted number of genets, excluding
non-beech sites (6 & 13)
m2 <- glm(formula = GenetsNr/SporocarpsNr ~ MeanAge,
          family = poisson(),
          data = tmeans[tmeans$Site[-c(6,13)],],
          weights = SporocarpsNr)
suppressWarnings(tab_model(m2))
```

	GenetsNr/SporocarpsNr		
Predictors	*Incidence Rate Ratios*	*CI*	*p*

(Intercept)	1.42	0.69 – 2.76	0.317
MeanAge	0.98	0.96 – 0.99	**0.001**
Observations	12		
R^2 Nagelkerke	0.687		

```
# build model for relationship between age of sites and weighted number of genets, excluding oak sites (6, 7 & 13)
m3 <- glm(formula = GenetsNr/SporocarpsNr ~ MeanAge,
          family = poisson(),
          data = tmeans[tmeans$Site[-c(6,7,13)],],
          weights = SporocarpsNr)
suppressWarnings(tab_model(m3))
```

	GenetsNr/SporocarpsNr		
Predictors	*Incidence Rate Ratios*	*CI*	*p*
(Intercept)	1.40	0.66 – 2.79	0.353
MeanAge	0.98	0.96 – 0.99	**0.001**
Observations	11		
R^2 Nagelkerke	0.685		

```
# build model for relationship between age of sites and weighted number of genets, including main tree species as factor
m4 <- glm(formula = GenetsNr/SporocarpsNr ~ MeanAge + MainSpecies,
          family = poisson(),
          data = tmeans,
          weights = SporocarpsNr)
suppressWarnings(tab_model(m4))
```

	GenetsNr/SporocarpsNr		
Predictors	*Incidence Rate Ratios*	*CI*	*p*
(Intercept)	1.41	0.66 – 2.80	0.344
MeanAge	0.98	0.96 – 0.99	**0.001**
MainSpecies: Birch	1.07	0.32 – 2.69	0.898
MainSpecies: Oak	12.78	1.05 – 128.58	**0.034**
Observations	14		
R^2 Nagelkerke	0.676		

```
# build model for relationship between age of sites and weighted number of genets, including "main tree species" as random effect
m5 <- lmer(formula = GenetsNr/SporocarpsNr ~ MeanAge + (1|MainSpecies),
           data = tmeans,
           weights = as.numeric(SporocarpsNr))
suppressWarnings(tab_model(m5))
```

	GenetsNr/SporocarpsNr		
Predictors	*Estimates*	*CI*	*p*
(Intercept)	0.78	0.28 – 1.28	**0.002**

MeanAge	-0.00	-0.01 – 0.00	0.060
**Random Effects**			
σ^2	0.85		
τ_{00} MainSpecies	0.03		
ICC	0.04		
N MainSpecies	3		
Observations	14		
Marginal R^2 / Conditional R^2	0.058 / 0.092		

```
# build model for relationship between age of sites and r, sMLH, Simpson's index,
# Shannon's H

m2 <- glm(formula = MeanAge ~ Shannon, data = tmeans)
m3 <- glm(formula = MeanAge ~ sMLH, data = tmeans)
m4 <- glm(formula = MeanAge ~ r, data = tmeans)
m5 <- glm(formula = MeanAge ~ Simpson, data = tmeans)
```

```
Shannon <- ggplot(data = tmeans, aes(x=Shannon, y=MeanAge)) +
  geom_point(aes(color= factor(Site), size= SporocarpsNr)) +
  scale_size_continuous(range = c(2, 8)) +
  geom_text_repel(aes(label=factor(Site)), segment.color = NA) +
  scale_color_viridis(discrete=TRUE, direction = -1, begin = 0.13) +
  theme_classic() +
  theme(text = element_text(size = 15),
        legend.position = "none") +
  labs(y = "", x = "Shannon's H") +
  geom_smooth(method = "glm", se = TRUE, color = "black") +
  scale_y_continuous(limits = c(-50,200)) +
  scale_x_continuous(breaks = pretty_breaks(5))

Simpson <- ggplot(data = tmeans, aes(x=Simpson, y=MeanAge)) +
  geom_point(aes(color= factor(Site), size= SporocarpsNr)) +
  scale_size_continuous(range = c(2, 8)) +
  geom_text_repel(aes(label=factor(Site)), segment.color = NA) +
  scale_color_viridis(discrete=TRUE, direction = -1, begin = 0.13) +
  theme_classic() +
  theme(text = element_text(size = 15),
        legend.position = "none") +
  labs(y = " ", x = "Simpson's index") +
  geom_smooth(method = "glm", se = TRUE, color = "black") +
  scale_y_continuous(limits = c(-50,200)) +
  scale_x_continuous(breaks = pretty_breaks(5))

sMLH <- ggplot(data = tmeans, aes(x=sMLH, y=MeanAge)) +
  geom_point(aes(color= factor(Site), size= SporocarpsNr)) +
  scale_size_continuous(range = c(2, 8)) +
  geom_text_repel(aes(label=factor(Site)), segment.color = NA) +
  scale_color_viridis(discrete=TRUE, direction = -1, begin = 0.13) +
  theme_classic() +
  theme(text = element_text(size = 15),
        legend.position = "none") +
  labs(y = "", x = "sMLH") +
  geom_smooth(method = "glm", se = TRUE, color = "black") +
  scale_y_continuous(limits = c(-50,200)) +
  scale_x_continuous(breaks = pretty_breaks(5))

r <- ggplot(data = tmeans, aes(x=r, y=MeanAge)) +
  geom_point(aes(color= factor(Site), size= SporocarpsNr)) +
  scale_size_continuous(range = c(2, 8)) +
  geom_text_repel(aes(label=factor(Site)), segment.color = NA) +
  scale_color_viridis(discrete=TRUE, direction = -1, begin = 0.13) +
  theme_classic() +
  theme(text = element_text(size = 15),
        legend.position = "none") +
  labs(y = "", x = "Relatedness") +
  geom_smooth(method = "glm", se = TRUE, color = "black") +
  scale_y_continuous(limits = c(-50,200)) +
  scale_x_continuous(breaks = pretty_breaks(5))

fig <- ggarrange(Shannon, Simpson, r, sMLH, ncol = 2, nrow = 2)
annotate_figure(fig,
                left = text_grob("Mean age (years)", rot=90, vjust = 1.5, size = 15))
```

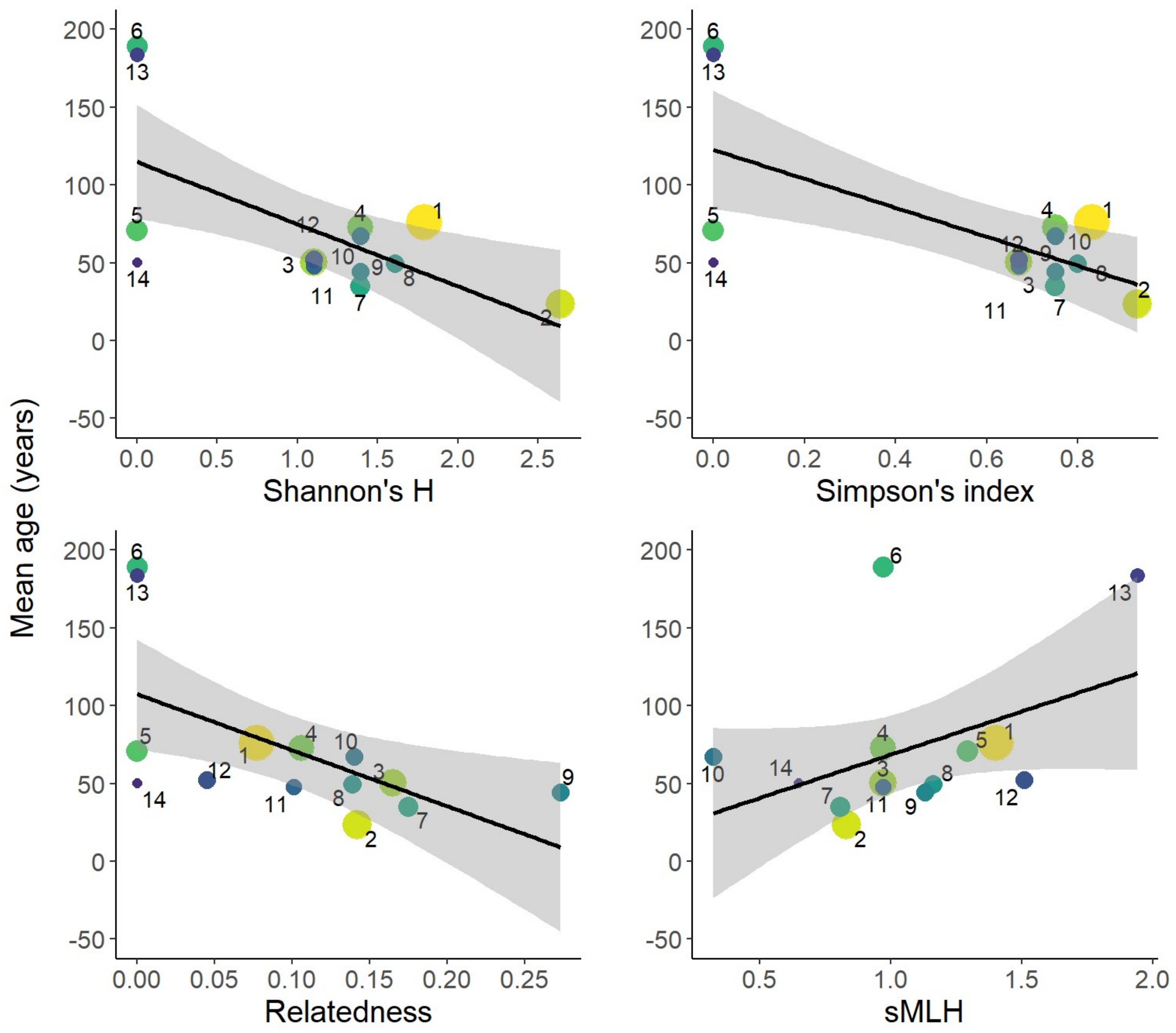
Mean age (years)
Shannon's H
Simpson's index
Relatedness
sMLH

***

```
## - Session info ---------------------------------------------------------------
##  setting  value
##  version  R version 3.5.3 (2019-03-11)
##  os       Windows 10 x64
##  system   x86_64, mingw32
##  ui       RTerm
##  language (EN)
##  collate  English_United States.1252
##  ctype    English_United States.1252
##  tz       Europe/Berlin
##  date     2020-07-02
##
## - Packages -------------------------------------------------------------------
##  package      * version  date       lib source
##  abind          1.4-5    2016-07-21 [1] CRAN (R 3.5.0)
##  ade4         * 1.7-13   2018-08-31 [1] CRAN (R 3.5.3)
##  adegenet     * 2.1.1    2018-02-02 [1] CRAN (R 3.5.3)
##  AICcmodavg   * 2.2-2    2019-05-29 [1] CRAN (R 3.5.3)
##  ape            5.3      2019-03-17 [1] CRAN (R 3.5.3)
##  assertthat     0.2.1    2019-03-21 [1] CRAN (R 3.5.3)
##  backports      1.1.4    2019-04-10 [1] CRAN (R 3.5.3)
##  bayestestR     0.4.0    2019-10-20 [1] CRAN (R 3.5.3)
##  boot           1.3-23   2019-07-05 [1] CRAN (R 3.5.3)
##  broom          0.5.2    2019-04-07 [1] CRAN (R 3.5.3)
##  car          * 3.0-3    2019-05-27 [1] CRAN (R 3.5.3)
##  carData      * 3.0-2    2018-09-30 [1] CRAN (R 3.5.2)
##  cellranger     1.1.0    2016-07-27 [1] CRAN (R 3.5.1)
##  class          7.3-15   2019-01-01 [2] CRAN (R 3.5.3)
##  classInt       0.4-1    2019-08-06 [1] CRAN (R 3.5.3)
##  cli            1.1.0    2019-03-19 [1] CRAN (R 3.5.3)
##  cluster        2.0.7-1  2018-04-13 [2] CRAN (R 3.5.3)
##  coda           0.19-3   2019-07-05 [1] CRAN (R 3.5.3)
##  codetools      0.2-16   2018-12-24 [2] CRAN (R 3.5.3)
##  colorspace     1.4-1    2019-03-18 [1] CRAN (R 3.5.3)
##  corrplot     * 0.84     2017-10-16 [1] CRAN (R 3.5.3)
##  cowplot        1.0.0    2019-07-11 [1] CRAN (R 3.5.3)
##  crayon         1.3.4    2017-09-16 [1] CRAN (R 3.5.1)
##  curl           4.0      2019-07-22 [1] CRAN (R 3.5.3)
##  data.table     1.12.2   2019-04-07 [1] CRAN (R 3.5.3)
##  DBI            1.0.0    2018-05-02 [1] CRAN (R 3.5.1)
##  deldir         0.1-23   2019-07-31 [1] CRAN (R 3.5.3)
##  digest         0.6.21   2019-09-20 [1] CRAN (R 3.5.3)
##  dplyr        * 0.8.3    2019-07-04 [1] CRAN (R 3.5.3)
##  e1071          1.7-2    2019-06-05 [1] CRAN (R 3.5.3)
##  emmeans        1.4.1    2019-09-12 [1] CRAN (R 3.5.3)
##  estimability   1.3      2018-02-11 [1] CRAN (R 3.5.2)
##  evaluate       0.14     2019-05-28 [1] CRAN (R 3.5.3)
##  expm           0.999-4  2019-03-21 [1] CRAN (R 3.5.3)
##  fansi          0.4.0    2018-10-05 [1] CRAN (R 3.5.3)
##  fastmatch      1.1-0    2017-01-28 [1] CRAN (R 3.5.2)
##  forcats        0.4.0    2019-02-17 [1] CRAN (R 3.5.3)
##  foreign        0.8-71   2018-07-20 [2] CRAN (R 3.5.3)
##  gdata          2.18.0   2017-06-06 [1] CRAN (R 3.5.1)
##  generics       0.0.2    2018-11-29 [1] CRAN (R 3.5.3)
##  ggeffects      0.12.0   2019-09-03 [1] CRAN (R 3.5.3)
##  ggplot2      * 3.3.2    2020-06-19 [1] CRAN (R 3.5.3)
##  ggpubr       * 0.2.3    2019-09-03 [1] CRAN (R 3.5.3)
##  ggrepel      * 0.8.1    2019-05-07 [1] CRAN (R 3.5.3)
##  ggsignif       0.6.0    2019-08-08 [1] CRAN (R 3.5.3)
##  glue           1.3.1    2019-03-12 [1] CRAN (R 3.5.3)
```

```
##  gmodels         2.18.1   2018-06-25 [1] CRAN (R 3.5.1)
##  gridExtra     * 2.3      2017-09-09 [1] CRAN (R 3.5.3)
##  gtable          0.3.0    2019-03-25 [1] CRAN (R 3.5.3)
##  gtools          3.8.1    2018-06-26 [1] CRAN (R 3.5.0)
##  haven           2.1.1    2019-07-04 [1] CRAN (R 3.5.3)
##  hms             0.5.1    2019-08-23 [1] CRAN (R 3.5.3)
##  htmltools       0.3.6    2017-04-28 [1] CRAN (R 3.5.1)
##  httpuv          1.5.2    2019-09-11 [1] CRAN (R 3.5.3)
##  httr            1.4.1    2019-08-05 [1] CRAN (R 3.5.3)
##  igraph          1.2.4.1  2019-04-22 [1] CRAN (R 3.5.3)
##  inbreedR      * 0.3.2    2016-09-09 [1] CRAN (R 3.5.3)
##  insight         0.6.0    2019-10-17 [1] CRAN (R 3.5.3)
##  kableExtra    * 1.1.0    2019-03-16 [1] CRAN (R 3.5.3)
##  KernSmooth      2.23-15  2015-06-29 [2] CRAN (R 3.5.3)
##  knitr           1.24     2019-08-08 [1] CRAN (R 3.5.3)
##  labeling        0.3      2014-08-23 [1] CRAN (R 3.5.0)
##  later           0.8.0    2019-02-11 [1] CRAN (R 3.5.3)
##  lattice         0.20-38  2018-11-04 [2] CRAN (R 3.5.3)
##  LearnBayes      2.15.1   2018-03-18 [1] CRAN (R 3.5.0)
##  lifecycle       0.1.0    2019-08-01 [1] CRAN (R 3.5.3)
##  lme4          * 1.1-21   2019-03-05 [1] CRAN (R 3.5.3)
##  magrittr      * 1.5      2014-11-22 [1] CRAN (R 3.5.3)
##  MASS            7.3-51.1 2018-11-01 [2] CRAN (R 3.5.3)
##  Matrix        * 1.2-15   2018-11-01 [2] CRAN (R 3.5.3)
##  mgcv            1.8-31   2019-11-09 [1] CRAN (R 3.5.3)
##  mime            0.7      2019-06-11 [1] CRAN (R 3.5.3)
##  minqa           1.2.4    2014-10-09 [1] CRAN (R 3.5.3)
##  mnormt          1.5-5    2016-10-15 [1] CRAN (R 3.5.2)
##  modelr          0.1.5    2019-08-08 [1] CRAN (R 3.5.3)
##  multcomp        1.4-10   2019-03-05 [1] CRAN (R 3.5.3)
##  munsell         0.5.0    2018-06-12 [1] CRAN (R 3.5.1)
##  mvtnorm         1.0-11   2019-06-19 [1] CRAN (R 3.5.3)
##  nlme            3.1-141  2019-08-01 [1] CRAN (R 3.5.3)
##  nloptr          1.2.1    2018-10-03 [1] CRAN (R 3.5.3)
##  openxlsx        4.1.0.1  2019-05-28 [1] CRAN (R 3.5.3)
##  parameters      0.2.0    2019-09-26 [1] CRAN (R 3.5.3)
##  pegas           0.11     2018-07-09 [1] CRAN (R 3.5.1)
##  performance     0.4.0    2019-10-21 [1] CRAN (R 3.5.3)
##  permute         0.9-5    2019-03-12 [1] CRAN (R 3.5.3)
##  phangorn        2.5.5    2019-06-19 [1] CRAN (R 3.5.3)
##  pillar          1.4.2    2019-06-29 [1] CRAN (R 3.5.3)
##  pkgconfig       2.0.2    2018-08-16 [1] CRAN (R 3.5.3)
##  plyr            1.8.4    2016-06-08 [1] CRAN (R 3.5.1)
##  polysat         1.7-4    2019-03-06 [1] CRAN (R 3.5.3)
##  poppr         * 2.8.3    2019-06-18 [1] CRAN (R 3.5.3)
##  promises        1.0.1    2018-04-13 [1] CRAN (R 3.5.1)
##  psych           1.8.12   2019-01-12 [1] CRAN (R 3.5.3)
##  purrr           0.3.2    2019-03-15 [1] CRAN (R 3.5.3)
##  quadprog        1.5-7    2019-05-06 [1] CRAN (R 3.5.3)
##  qvalue        * 2.15.0   2019-10-09 [1] Github (jdstorey/qvalue@9b3f9a8)
##  R6              2.4.0    2019-02-14 [1] CRAN (R 3.5.3)
##  raster          3.0-2    2019-08-22 [1] CRAN (R 3.5.3)
##  Rcpp            1.0.2    2019-07-25 [1] CRAN (R 3.5.3)
##  readr           1.3.1    2018-12-21 [1] CRAN (R 3.5.2)
##  readxl        * 1.3.1    2019-03-13 [1] CRAN (R 3.5.3)
##  reshape2      * 1.4.3    2017-12-11 [1] CRAN (R 3.5.3)
##  rio             0.5.16   2018-11-26 [1] CRAN (R 3.5.3)
##  rlang           0.4.0    2019-06-25 [1] CRAN (R 3.5.3)
##  rmarkdown       2.2      2020-05-31 [1] CRAN (R 3.5.3)
##  rstudioapi      0.10     2019-03-19 [1] CRAN (R 3.5.3)
```

```
##  rvest           0.3.4    2019-05-15 [1] CRAN (R 3.5.3)
##  sandwich        2.5-1    2019-04-06 [1] CRAN (R 3.5.3)
##  scales        * 1.0.0    2018-08-09 [1] CRAN (R 3.5.3)
##  seqinr          3.6-1    2019-09-07 [1] CRAN (R 3.5.3)
##  sessioninfo     1.1.1    2018-11-05 [1] CRAN (R 3.5.3)
##  sf              0.7-7    2019-07-24 [1] CRAN (R 3.5.3)
##  shiny           1.3.2    2019-04-22 [1] CRAN (R 3.5.3)
##  sjlabelled      1.1.1    2019-09-13 [1] CRAN (R 3.5.3)
##  sjmisc          2.8.2    2019-09-24 [1] CRAN (R 3.5.3)
##  sjPlot        * 2.7.2    2019-09-29 [1] CRAN (R 3.5.3)
##  sjstats         0.17.6   2019-09-08 [1] CRAN (R 3.5.3)
##  sp              1.3-1    2018-06-05 [1] CRAN (R 3.5.1)
##  spData          0.3.0    2019-01-07 [1] CRAN (R 3.5.3)
##  spdep           1.1-2    2019-04-05 [1] CRAN (R 3.5.3)
##  stringi         1.4.3    2019-03-12 [1] CRAN (R 3.5.3)
##  stringr         1.4.0    2019-02-10 [1] CRAN (R 3.5.3)
##  survival        2.43-3   2018-11-26 [2] CRAN (R 3.5.3)
##  TH.data         1.0-10   2019-01-21 [1] CRAN (R 3.5.3)
##  tibble          2.1.3    2019-06-06 [1] CRAN (R 3.5.3)
##  tidyr         * 1.0.0    2019-09-11 [1] CRAN (R 3.5.3)
##  tidyselect      0.2.5    2018-10-11 [1] CRAN (R 3.5.2)
##  units           0.6-4    2019-08-22 [1] CRAN (R 3.5.3)
##  unmarked        0.12-3   2019-02-05 [1] CRAN (R 3.5.3)
##  utf8            1.1.4    2018-05-24 [1] CRAN (R 3.5.1)
##  vctrs           0.2.0    2019-07-05 [1] CRAN (R 3.5.3)
##  vegan           2.5-6    2019-09-01 [1] CRAN (R 3.5.3)
##  VGAM            1.1-1    2019-02-18 [1] CRAN (R 3.5.3)
##  viridis       * 0.5.1    2018-03-29 [1] CRAN (R 3.5.1)
##  viridisLite   * 0.3.0    2018-02-01 [1] CRAN (R 3.5.1)
##  webshot         0.5.1    2018-09-28 [1] CRAN (R 3.5.3)
##  withr           2.1.2    2018-03-15 [1] CRAN (R 3.5.1)
##  xfun            0.9      2019-08-21 [1] CRAN (R 3.5.3)
##  xml2            1.3.2    2020-04-23 [1] CRAN (R 3.5.3)
##  xtable          1.8-4    2019-04-21 [1] CRAN (R 3.5.3)
##  yaml            2.2.0    2018-07-25 [1] CRAN (R 3.5.1)
##  zeallot         0.1.0    2018-01-28 [1] CRAN (R 3.5.3)
##  zip             2.0.4    2019-09-01 [1] CRAN (R 3.5.3)
##  zoo             1.8-6    2019-05-28 [1] CRAN (R 3.5.3)
##
## [1] C:/Users/localadmin/Documents/R/win-library/3.5
## [2] C:/Program Files/R/R-3.5.3/library
```

---

1. Kamvar, Z.N., Tabima, J.F., Gruenwald, N.J. (2014) Poppr: an R package for genetic analysis of populations with clonal, partially clonal, and/or sexual reproduction. PeerJ 2:e281. doi: 10.7717/peerj.281.↩

2. Kamvar, Z.N., Brooks, J.C. and Gruenwald, N.J. (2015) Novel R tools for analysis of genome-wide population genetic data with emphasis on clonality. Front. Genet. 6:208. doi: 10.3389/fgene.2015.00208↩

3. Peakall, R. and Smouse P.E. (2012) GenAlEx 6.5: genetic analysis in Excel. Population genetic software for teaching and research-an update. Bioinformatics 28, 2537-2539.↩

4. Lynch & Ritland (1999) Estimation of pairwise relatedness with molecular markers. Genetics. 152(4):1753-66↩

5. Wei, T. and Simko, V. (2017). R package "corrplot": Visualization of a Correlation Matrix (Version 0.84). Available from https://github.com/taiyun/corrplot (https://github.com/taiyun/corrplot)↩

6. Stoffel, M. A., Esser, M., Kardos, M., Humble, E., Nichols, H., David, P., & Hoffman, J. I. (2016). inbreedR: An R package for the analysis of inbreeding based on genetic markers. Methods in Ecology and Evolution.↩
